# Extra-abdominal infections caused by *Comamonas kerstersii*: Case report

**DOI:** 10.1097/MD.0000000000037161

**Published:** 2023-02-02

**Authors:** Mingxi Wang, Hui Wang, Shicheng Chen, Desong Ming, Qingbin Nie

**Affiliations:** a Yun Leung Laboratory for Molecular Diagnostics, School of Medicine, Huaqiao University, Xiamen, Fujian, China; b Medical Laboratory Science, School of Health Sciences, Northern Illinois University, Dekalb, IL, USA; c Desong Ming, Department of Clinical Laboratory, Quanzhou First Hospital Affiliated to Fujian Medical University, Fujian, China; d Department of Neurosurgery, Chinese PLA General Hospital, Beijing, 100853, China.

**Keywords:** case report, *Comamonas kerstersii*, extra-abdominal infection, extensive drug resistance, pneumonia

## Abstract

**Rationale:**

*Comamonas kerstersii* mainly causes intra-abdominal infections with favorable outcomes due to high antibiotic susceptibility. We report the first case of pneumonia caused by *C Kerstersii*, which promoted patient death, and a second urinary tract infection by *C Kerstersii* with extensive drug resistance.

**Patient concerns:**

A 46-year-old male (Case 1) with craniocerebral injury underwent emergency decompressive craniectomy, but his condition deteriorated further and presented with discontinuous fever, small moist rales on both lungs, and respiratory failure. Retrospective average nucleotide identity (ANI) analysis of the genomic sequence of the sputum isolate identified it as *C Kerstersii* 12322-1, antimicrobial susceptibility testing (AST) revealed that it was sensitive to 18 of 21 tested antibiotics.

An 82-year-old male (Case 2) with hypertrophic prostate experienced gradual obstruction during urination, and a urine test revealed WBC ++. Retrospective ANI analysis of the urine isolate identified it as *C Kerstersii* 121606, which was resistant to 18 of 21 tested antibiotics.

**Diagnoses:**

Case 1 was diagnosed empirically as pneumonia caused by *C Kerstersii* strain 12322-1 secondary to craniocerebral injury and confirmed by retrospective ANI analysis; case 2 was diagnosed empirically as urinary infection secondary to prostate hyperplasia caused by *C Kerstersii* strain 121606 confirmed by the retrospective ANI analysis.

**Interventions:**

Case 1 was administered cefoxitin, cefodizime, imipenem-cilastatin sodium, and underwent comprehensive salvage management. Case 2 was administered doxycycline alone.

**Outcomes:**

Case 1 died partially because of untimely identification of the responsible bacteria-12322-1. Case 2 was cured even 121606 exhibited an extensive drug resistance feature.

**Lessons:**

Except for intra-abdominal infections with good prognosis, we verified that *C Kerstersii* could also cause extra-abdominal infections, such as the first pneumonia case and urinary infection. It could promote patient death; actual infections were underestimated due to identification difficulties, posing a health threat due to the presence of extensive drug resistance.

## 1. Introduction

*Comamonas* spp. are gram-negative rods that can be found throughout the environment, including in water, soil, plants, and animals.^[1–3]^ Although generally thought to have low virulence, a few *Comamonas* species (especially *C testosterone, C kerstersii*) can still cause opportunistic infections in immunocompetent hosts.^[2,4]^ *C kerstersii* was first identified in 2003,^[4]^ and since has been predominantly associated with intra-abdominal infections, including appendicitis, peritonitis, diverticulosis.^[2]^ 27 *C kerstersii* strains have also been cultured from human fecal samples, suggesting a possible link with the gastrointestinal tract.^[5]^ The majority of *C kerstersii* infections appear to be polymicrobial, with 1 case of urinary tract infection being an exception.^[6]^ *C kerstersii* isolates have shown high susceptibility to antibiotics.^[2,7,8]^ Consequently, the management of *C kerstersii* infections with appropriate antimicrobial therapy often results in favorable clinical outcomes, even in cases of mixed infections^[1,5,8]^

However, in our clinical study, we encountered a serious challenge posed by *C Kerstersii*. It caused not only intra-abdominal infections-acute perforated appendicitis, but also extra-abdominal infections-lung infection, which promoted the death of the patient, and urinary tract infection, which presented an extensive drug resistance (XDR) phenotype. Moreover, the actual number of clinical infections caused by *C Kerstersii* was seriously underestimated, as all of our *C Kerstersii* strains were misidentified by biochemical identification methods and even by 16S rRNA sequencing. Here, we present the first case of pneumonia and the second case of urinary tract infection caused by *C Kerstersii* to emphasize the need for healthcare professionals to reassess the potential health risks posed by *C kerstersii*.

## 2. Case presentation

### 2.1. Case 1

A 46-year-old male presented to the emergency department with traumatic craniocerebral injury and coma for 4 hours following a traffic accident. Upon admission, head computed tomography (CT) performed at another hospital revealed an epidural hematoma beneath the left temporal and parietal skull, along with a cerebral hernia and subarachnoid hemorrhage (SAH). During physical examination, the patient Glasgow Coma Scale score was 4, indicating severe impairment of consciousness. Examination of the pupils revealed the absence of direct and indirect light reflection in the right (4 mm diameter) and left pupils (5 mm diameter). Furthermore, the patient exhibited hyperextension, rigidity, hypertonia, and hypertendinous and Babinski reflexes in the extremities. These findings suggest significant neurological impairment. Laboratory tests showed hyponatremia (Na^+^, 128 mmol/L) and an abnormal blood test result, with a white blood cell count (WBC) of 11.1 × 10^9^/L and a high neutrophil percentage of 91.40%. The patient was diagnosed with an acute severe closed craniocerebral injury with cerebral hernia, SAH, left temporoparietal epidural hematoma, and scalp contusion.

To address his condition, the patient underwent decompressive craniectomy using a left frontotemporal approach. During surgery, it was discovered that the left middle dural artery ruptured, resulting in intracranial bleeding. Additionally, a left temporoparietal epidural hematoma, approximately 70 mL in size, and SAH were observed as shown on the CT scan. Furthermore, rupture and hemorrhage of the cortical venous vessels, punctate contusion, and laceration of the left temporoparietal brain were observed. Bleeding was stabilized using the double-pole electric-coagulative hemostasis method. After the surgery, a hemostatic [tranexamic acid, 1.0 g, twice a day (bid) from Day 1 to Day 2 postsurgery (abbreviated as D1_PS-D3_PS)], a dehydrant [mannitol 125 ml, iv drip, every 6 hours (Q6h), D1_PS-D2_PS; then 125 ml, iv drip, Q12h, D2_PS], an inhibitor of brain edema [dexamethasone 10 mg, iv drip, twice per day (bid), D1_PS-D5_PS; then 5 mg, iv drip, bid, D5_PS-D7_PS], diuretics (furosemide 20 mg, Q8h, D1_PS-D5_PS, then 80 mg on D9_PS and 20 mg per day on D10_PS-D11_PS), and preventive agents for stress-induced gastrointestinal ulcer (famotidin 40 mg, iv drip, bid, D1_PS-D5_PS, then omeprazole sodium 40 mg, iv drip, Qd, D5_PS-D23_PS) were given.

Management of the respiratory and urinary tracts, parenteral nutrition support, monitoring of vital signs, blood oxygen saturation (SPO_2_), blood analysis, blood electrolytes, glucose, and biochemical tests for liver and renal function were continuously performed. The patient Glasgow Coma Scale did not improve from 4 or 5, and he remained unconscious until his death on D23_PS. Prior to his death, his general condition progressed drastically, further complicated by intermittent fever (D1_PS-D23_PS), pneumonia (diagnosed on D1_PS, based on the presentation of small moist rales heard on both lung bases, fever and respiratory failure, aggravated and maintained until death), and respiratory failure (diagnosed on D4_PS and maintained until death), hypertension (215/68 mm Hg on D4_PS), hypotension (105/56 mm Hg on D8_PS, 78/49 mm Hg on D9_PS, lasting until death), hyponatremia (Na^+^ 128 mmol/L on D1_PS), acute renal insufficiency {diagnosed on D1_PS [creatinine (CR) 226 μmol/L], aggravated on D2_PS (CR 605 μmol/L)} and renal failure (diagnosed on D3_PS)}, hyperkalemia [diagnosed on D5_PS (K^+^ 5.60 mmol/L), aggravated on D7_PS (K^+^ 5.90 mmol/L), lasted until D10_PS (K^+^:5.90–7.40 mmol/L)], severe anemia {diagnosed on D1_PS [red blood cell (RBC) 2.55 × 10^12^/L; hemoglobin (HGB) 82 g/L], aggravated until death [RBC: (1.79–2.35) × 10^12^/L; HGB:56-74 g/L]}, thrombocytopenia {diagnosed on D1_PS [platelet (PLT) 86 × 10^9^/L], aggravated until death [PLT: (50–96) × 10^9^/L]}, impaired liver function {diagnosed on D1_PS [aspartate aminotransferase 74 IU/L, alanine aminotransferase (ALT) 67 IU/L]}, aggravated on D8_PS (ALT 283 IU/L)], hypoproteinemia [diagnosed on D1_PS, lasted at least until D16_PS (albumin 11.7–26 g/L)], hyperglycemia [diagnosed on D1_PS, lasted at least until D13_PS (7.43–15.8) mmol/L], diarrhea (diagnosed on D14_PS), and epilepsy (diagnosed on D9_PS). The patient underwent tracheal intubation ventilator-assisted breathing (D5_PS-D22_PS) and other supportive management, such as blood dialysis.

Six days after death, the patient sputum was cultured successfully, and the BD Phoenix automated identification with the NMIC/ID-4 panel identified the bacterium, *Delftia acidovorans*. Antimicrobial susceptibility testing (AST) revealed that it was sensitive to 18 of the 21 antibiotics included in the panel (NMIC/ID-4), except for aztreonam, ciprofloxacin, and tetracycline. Later, retrospective 16S rRNA gene sequencing and Blast analysis mistakenly identified as *Acidovorax* spp. (1396 bp, GenBank accession no. KR349265.1). However, its final identity as *C kerstersii* (strain 12322-1) was confirmed through average nucleotide identity (ANI) analysis of its genomic sequence (unpublished data). Because of late successful culturing and misidentification, these data did not have the opportunity to show substantive promotion of treatment.

### 2.2. Case 2

Upon admission, an 82-year-old male patient presented with a 3-year history of gradual obstruction during urination. Routine urine examination revealed WBC ++ and occult blood cell counts +. Abdominal color ultrasound revealed bilateral renal cysts, residual urine in the bladder, a hypertrophic prostate, and calcified prostate lesions. After a thorough examination, a diagnosis of prostate gland hyperplasia complicated by urinary tract infection was established.

The urine sample was cultured, and the BD Phoenix automated identification system identified bacterium-*Sphingobacterium multivorum* (with 92% confidence). Retrospective 16S rRNA gene sequencing and Blast analysis mistakenly identified it as *Acidovorax* spp. (1460 bp, GenBank accession no. KY014106). Its final identity as *C kerstersii* (strain 121606) was verified using ANI analysis of its genomic sequence (unpublished data).

AST revealed that strain 121606 was resistant to 18 of the 21 tested antibiotics, including aminoglycosides (amikacin and gentamicin), nearly all β-lactams {penicillins (ampicillin, piperacillin, amoxicillin/clavulanic acid, ampicillin/sulbactam, piperacillin/tazobactam), cephalosporins (cefazolin, ceftazidime, cefotaxime, and cefepime), carbapenems (imipenem), monobactams (aztreonam)}, chloramphenicols (chloramphenicol), polypeptides (colistin), quinolones (ciprofloxacin, levofloxacin, moxifloxacin), and sulfonamides (trimethoprim/sulfamethoxazole), it was intermediately sensitive to piperacillin/tazobactam, and sensitive to tetracycline and meropenem. These data demonstrate that *C kerstersii* 121606 possesses a XDR phenotype.

## 3. Treatment

### 3.1. Case 1

The patient received a course of antimicrobial drugs to treat the pneumonia and prevent other potential infections. The prescribed regimen included cefoxitin sodium (2.0 g, iv drip, bid, D1_PS-D3_PS), cefodizime sodium (1.0 g iv drip, D3_PS-D7_PS), and imipenem-cilastatin sodium (0.5 g, iv drip, D10_PS-D16_PS). The patient died of cardiac arrest and absence of spontaneous breathing on D23_PS after 24 days of hospitalization.

### 3.2. Case 2

Based on the AST results, this patient was administered only antimicrobial therapy (doxycycline) to treat urinary tract infection secondary to prostate gland hyperplasia due to comorbid heart dysfunction, atrial fibrillation, frequent premature ventricular contraction, and ST-T change, all of which were contraindications to prostate surgery. The patient had no symptoms of urinary system infection following antimicrobial therapy or during the follow-up.

## 4. Discussion

The predominance of intra-abdominal infections caused by *C kerstersii*^[7,9,10]^ indicates that the gastrointestinal tract is a habit. To date, only 2 cases of extra-abdominal infections caused by *C kerstersii* have been reported: urinary tract infection^[6]^ and bacteremia.^[11]^ We present the second reported case of urinary tract infection (Case 2) and the first case of lung infection (Case 1) caused by *C kerstersii.* These cases demonstrate the importance of identifying potential extra-abdominal pathogenicity of *C kerstersii*.

Early and accurate identification of this bacterium, along with appropriate antimicrobial treatment, is crucial for effective management of *C kerstersii* infections. In Case 1, the patient had persistent and severe pneumonia caused by strain 12322-1. Even strain 12322-1 was sensitive to most of the tested antibiotics; however, this bacterium was not successfully cultured and was correctly identified early by conventional biochemical identification methods, leading to aimless antibiotic application and promoting the patient death. During the identification process of *C kerstersii*, we encountered the same difficulty in identifying another strain (202149), as in the identification process of strain 12322-1 in Case 1 and strain 121606 in Case 2. Strain 202149 was isolated from peritoneal pus culture of a 12-year-old girl with acute perforated appendicitis. It was misidentified as *C testosteroni* (with 98% confidence) by the BD Phoenix automated identification system and mistakenly identified as *Acidovorax* spp. by BlastN analysis of its 16S rRNA gene sequence (1462 bp, GenBank accession No. KY012351), confirmed as *C kerstersii* by ANI analysis. All of these difficulties in isolation and identification by conventional biochemical phenotyping methods lead to untimely or incorrect antibiotic application and our patient death to some extent.

These phenotypic identification methods have historically faced challenges in accurately distinguishing *Comamonas* species from closely related species^[7]^ and resulting in serious underestimation of actual *C kerstersii* infections until the introduction of matrix-assisted laser desorption ionization time-of-flight mass spectrometry.^[3]^ Matrix-assisted laser desorption ionization time-of-flight mass spectrometry has emerged as the preferred choice because of its quick turnaround, accuracy, and ease of use in distinguishing closely related species.^[11,12]^ For *Comamonas* species, 16S rRNA gene sequencing, the gold standard bacterial identification method, could not classify them as mentioned above. Although genomic sequencing analysis has shown promise for bacterial identification, it is not yet a routine laboratory method for bacterial identification.^[3]^

Case 2 involved a patient with urinary tract infection caused solely by strain 121606. However, strain 121606 demonstrated XDR, indicating potential difficulties in treating *C kerstersii* infections, even though case 2 was cured. A XDR *C kerstersii* has not been reported yet.

## 5. Conclusion

*C kerstersii* mainly causes intra-abdominal infections and is highly sensitive to antibiotics. However, we provide evidence that *C kerstersii* could also cause fatal pneumonia and exhibit XDR, and its infections are seriously underestimated. It is crucial to take urgent measures to comprehend the mechanisms underlying XDR and pathogenicity to develop effective treatment strategies and control the spread of infections caused by this pathogen. For this purpose, we performed genomic sequencing of these isolates and comparative genomic analysis to understand XDR mechanisms and pathogenicity in the lungs and gastrointestinal tract.

## Author contributions

**Conceptualization:** Mingxi Wang.

**Data curation:** Mingxi Wang, Hui Wang, Desong Ming, Qingbin Nie.

**Formal analysis:** Mingxi Wang, Hui Wang, Shicheng Chen, Desong Ming, Qingbin Nie.

**Funding acquisition:** Mingxi Wang, Qingbin Nie.

**Investigation:** Mingxi Wang, Hui Wang, Shicheng Chen, Desong Ming.

**Methodology:** Mingxi Wang, Hui Wang, Desong Ming, Qingbin Nie.

**Project administration:** Mingxi Wang, Qingbin Nie.

**Resources:** Mingxi Wang, Qingbin Nie.

**Supervision:** Mingxi Wang.

**Validation:** Mingxi Wang, Shicheng Chen, Desong Ming, Qingbin Nie.

**Visualization:** Mingxi Wang.

**Writing – original draft:** Mingxi Wang, Hui Wang, Shicheng Chen, Qingbin Nie.

**Writing – review & editing:** Mingxi Wang, Shicheng Chen, Desong Ming, Qingbin Nie.
